# Development and evaluation of passenger assistance system concepts to reduce passenger discomfort

**DOI:** 10.3389/fpsyg.2023.1024540

**Published:** 2023-02-09

**Authors:** Sandra Ittner, Dominik Mühlbacher, Mark Vollrath, Thomas H. Weisswange

**Affiliations:** ^1^Würzburg Institute for Traffic Sciences (WIVW) GmbH, Veitshöchheim, Germany; ^2^Technische Universität Braunschweig, Braunschweig, Germany; ^3^Honda Research Institute Europe GmbH, Offenbach, Germany

**Keywords:** passenger, simulator study, discomfort, assistance systems, human-machine interface

## Abstract

The front seat passenger is often neglected when developing support systems for cars. There exist few examples of systems that provide information or interaction possibilities specifically to those passengers. Previous research indicated that the passive role of the passenger can frequently lead to a feeling of discomfort, potentially caused by missing information and missing control with respect to the driving situation. This paper investigates if and how different aspects of cognitive processes as defined in a previously published model can be approached with a technical system to reduce discomfort in passengers. Five prototypical passenger assistance systems are created which provide missing information (for example about the attentiveness of the driver) or the possibility to have more influence as a passenger. In a static simulator study with *N* = 40 participants, these systems were investigated with respect to their influence on measures of discomfort. Participants experienced in a counterbalanced order car following and braking scenarios on the highway with different time headways (within-subjects), with and without one of the passenger assistance systems (between-subjects). Based on the subjective measures for each experienced situation, three systems were identified as particularly useful in reducing discomfort. These displayed the attentiveness of the driver, the safety distance to a vehicle in front or provided the possibility to signal the driver that the recent safety distance is too small. These best proposals significantly reduced passenger discomfort in the tested Following and Braking scenarios for different time headways. In the post inquiry, more than 64% of the passengers confirmed the helpfulness of the rated system in reducing their discomfort in each case and about 75% of the passengers reported an interest in using it in their vehicle. This demonstrates opportunities to improve the everyday driving experience beyond classical assistance systems by explicitly considering the needs of passengers.

## 1. Introduction

Many people know the feeling of being a front seat passenger, experiencing situations in which they would have wished to have access to their own brake pedal, for example when a driver is distracted while following too closely. Such events usually result in a feeling of discomfort. The Oxford English Dictionary (Stevenson and Lindberg, 2017) defines “discomfort” as a feeling of “slight pain” or “to make (someone) feel uneasy, anxious or embarrassed.” The definition of discomfort shows a connection to emotions like fear and to social emotions like embarrassment. Similar to these emotions, feelings of discomfort are a result of the evaluation of internal and external stimuli and signal motivational significance (Bower and Cohen, 1982; Lazarus, 1982; Leventhal and Scherer, 1987). Discomfort can be caused by different circumstances in the environment or in the person experiencing it such as uncomfortable seats, interactions between humans, motion sickness, or the cognitive evaluation processes of environmental situations during driving. For a more detailed discussion on the definition, the reader is referred to Ittner et al. (2020).

This study focuses on discomfort experienced by a passenger, as an outcome to the perceived criticality or uncertainty of a dynamic situation. An example would be discomfort felt by a passenger when the driver, in the passenger’s opinion, overtakes in a crowded situation or does not pay sufficient attention to a potentially critical traffic participant. Previous interview and questionnaire studies (Ittner et al., 2020) showed that 88% of passengers experienced an uncomfortable situation at least once, frequently caused by fast driving and close following. As feeling discomfort is caused by a subjective evaluation of the circumstances, it can be triggered either by objectively critical situations, where it could be considered an appropriate warning signal of the body, or by a misjudgment of situational aspects. Objectively safety-critical situations would include, for example, situations in which the driver follows another car with less than the legal minimum distance, but also situations in which the driver would be distracted for a longer period, as in the case of cell phone use while driving. As described in previous work (Ittner et al., 2020), discomfort caused by such situation can also turn into a feeling of anxiety. Anxiety is an emotion in reaction to a situation that is considered dangerous or harmful, for example, because the driver no longer has control over the situation. This work aims to support the passenger with the help of an assistance system to assess objectively still safe situations, which are wrongly assessed as safety critical by the passenger due to missing information or control. The focus is on selecting and addressing situations that do not (yet) cause anxiety in most passengers. In most traffic situations a driver will also assess the situation. If (s) he would consider it critical or uncomfortable, one would expect an adaptation of the vehicle controls to improve the situation if possible. In the above examples of close following and fast driving, this would mean slowing down or increasing the distance. If the driver does not react but the passenger still feels uncomfortable, there might be a misjudgment by the latter, which will be the scenario considered in this paper. Based on these considerations and well-established psychological models, like the feedback-loop model by Carver and Scheier (2002) and the transactional stress model by Lazarus and Folkman (1984), a cognitive model of causes for passenger discomfort was proposed in Ittner et al. (2020). This model tries to explain why passengers experience discomfort in situations that are subjectively evaluated as not critical by the driver. The model focuses on two main differences that could explain passenger discomfort while the driver is not experiencing discomfort. Firstly, there exists a difference in the estimation of the situation between driver and passenger caused by limited or missing information. Secondly, the passenger has no direct control over the vehicle as (s) he does not have access to means to, for example, increase the distance to a vehicle in front or to reduce speed. The model provides multiple distinct information pathways that could contribute to these two aspects.

If the reason for the asymmetry in discomfort between driver and passenger would be known, one could think about means to prevent or reduce it. Approaching such means can also further validate specific pathways in the model. A technical way to try to resolve problems of missing information or interaction possibilities is the introduction of human-machine interfaces or assistance systems that find ways to provide these. While there is ample research on the positive effects of human-machine interfaces and assistance systems for drivers of vehicles, it has not yet been studied for passengers. This paper will investigate if similar concepts can be used to improve the driving experience of passengers. However, the main factor controlling the driving process in a passenger setting is the driver, which introduces additional challenges for providing relevant information in a human-machine interface. This work aims to investigate potential sources of information and evaluate various human-machine interface concepts for passenger assistance systems with a focus on reducing the feeling of discomfort in specific, non-critical situations. Five concepts are developed based on the cognitive passenger discomfort model proposed in Ittner et al. (2020) and tested in a user study in the simulator.

The next section will discuss existing approaches for assistance in vehicles from the areas of driver assistance and automated driving research and look at concepts that could be adapted for passenger human-machine interfaces. After this, the cognitive passenger discomfort model will be briefly re-introduced and used to develop concepts for passenger assistance to reduce discomfort. To test the concepts with users, a small feasibility study first establishes a simulator setting that works for passengers and is able to trigger feelings of discomfort in participants. With this setup, a user study with 40 participants evaluates the human-machine interface concepts with respect to their acceptance and potential of reducing discomfort reports of participants. The paper will close with a discussion of the results, possible limitations and open questions.

## 2. Related work

So far, there is little work on assistance systems dedicated to support passengers of motor vehicles. Generally, the passenger has played only a small or no role in the development of human-machine interfaces for road vehicles and there are only a few human-machine interfaces specifically designed for them. Most of these systems focus on infotainment (e.g., Meixner et al., 2017; Sen and Sener, 2020) or on information for the passenger which help to support the driver (Maurer et al., 2014; Perterer et al., 2015; Trösterer et al., 2015, 2019). As the former is usually independent of the current driving situation, this section will focus on research about the latter. In studies by Maurer et al. (2014) and Trösterer et al. (2015), the front seat passenger’s gaze is visualized in a driving simulation to improve communication and avoid misunderstandings with the driver during demanding situations like navigation or upcoming hazards. Supporting the passenger to take over tasks like navigation is intended to relieve the driver and their evaluation therefore focused mostly on driver benefits. Both systems, as well as a simplified version where an LED strip was attached along the bottom of the windshield which was tested in a real vehicle (Trösterer et al., 2019), could be shown to receive positive ratings regarding helpfulness for the driver, driving performance, and communication accuracy. Perterer et al. (2015) investigated the influence of a more detailed, dedicated navigation display for the passenger to take care of the navigation task. The system showed additional information such as upcoming hazards. Most of the drivers of a user study (75%) described the support by the passenger using the assistance system as relieving. However, none of the studies evaluated the effect on passenger satisfaction or discomfort, and the proposed human-machine interfaces did not aim at providing information or other means to directly improve the driving experience of the passenger. In a parallel study, Ittner et al. (2021) evaluated an explicit instance of such an assistance system with users on real highways. They used an LED interface to inform both driver and passenger of the objective appropriateness of distances to a front vehicle. This design was based on the same theoretical considerations that will be used in this paper. However, testing in real world traffic implied limitations with respect to the repeatability of the situations and required compromises in the setup of the experiments and the implementation of the assistance system.

Human-machine interfaces for driver assistance are well established and have been investigated in detail in many studies so far (Adell et al., 2008; Charissis and Papanastasiou, 2010; Hofauer et al., 2018; Rittger and Götze, 2018; Winkler et al., 2018; Aydogdu et al., 2019; Schick et al., 2019). The goal of such human-machine interfaces is to support the driver by providing different types of information that help him/her in performing the driving task and to avoid unwanted situations. This can also have a positive impact on the comfort of drivers, although most studies only evaluate related concepts like stress, safety feeling, or perceived transparency of a situation. As an example, in a Study by Charissis and Papanastasiou (2010), a human-machine interface highlighted vehicles on the road, increasing spatial awareness and reducing response times under low visibility conditions. They could show that participants had fewer collisions using the system, an improved response time, and 90% of the participants stated that the system reduced their stress during the low visibility conditions. An information mismatch between what the driver perceives and how this is reflected in situational control is introduced through the advent of advanced driver assistance systems such as adaptive cruise control or lane keeping assistance that actively support the driver in vehicle control. Addressing this, human-machine interfaces are also used to visualize processes of active driver assistance systems which are not directly visible to the driver. Aydogdu et al. (2019) compared a human-machine interface showing information about a lane keeping assistance system deployment and situation understanding explicitly through a head-up display with a commercial display. In a pilot study (Schick et al., 2019), they showed that the conventional design caused a lack of transparency and made the participants feel stressed and not safe. In contrast, in the following public road user study (Aydogdu et al., 2019) participants reported increased system transparency, increased safety, and decreased monitoring effort with the head-up display. This shows that even making existing information more available can have a positive effect on driver emotions. The exact reasons for stress and mistrust might differ between passengers and drivers, however, the concept of increased transparency of driving information might be transferrable.

In the area of automated driving the information mismatch for the driver is even more evident as almost all driving processes are not directly accessible by the driver. This is similar to the situation of a passenger in manually driven vehicles for whom the performance of the driving task by the driver is also only partially visible. Besides the purpose to show information about a vehicle’s system status, automated driving human-machine interfaces can also communicate more advanced information like planned maneuvers or system limits to prepare the driver to regain control. Many studies in this area focus their evaluation on trust rather than comfort but situational trust shares many aspects with psychological discomfort (Hoff and Bashir, 2014). Chang et al. (2019) investigated the influence of different amounts of information on trust in an automated taxi. They visualized information about traffic controls, the intended path of the automated vehicle, or about other road users. Participants trusted the automated vehicle more when receiving this information. This relationship between system transparency and increased user trust was also confirmed by other studies [e.g., Dzindolet et al. (2003) or Kaltenbach and Dolgov’s (2017) overview in Hoff and Bashir (2014)]. Besides possible asymmetries in information processing, the “driver” of a fully automated vehicle has limited influence on vehicle behavior, which is another similarity to passengers in conventional vehicles. Automated driving human-machine interfaces tried to improve driving experience by directly adapting path planning to driver comfort (Elbanhawi et al., 2015), but more often were proposed as dedicated ways to influence the driving process. A study by Frison et al. (2017) showed that providing a small level of voluntary control significantly improved positive feelings of participants experiencing otherwise fully automated driving. However, there are also two studies in this area that show a direct positive effect of driving-related information conveyed with the help of an human-machine interface on safety, understanding and driving comfort of the driver during automated driving (Hartwich et al., 2021). In another study, it was shown that perceived safety and comfort were higher when trust in the system was higher. When trust was lower, drivers wanted to be provided with more information during the ride (Hartwich et al., 2020). These results suggest that with the help of human-machine interfaces, in addition to a transparent presentation of information, the provision of influence or control can have a positive impact on the driving experience. Due to the similarities between automated driving drivers and passengers, the reported human-machine interface concepts might provide helpful insights for the design of passenger assistance systems.

Although there are similarities between passengers in manually driven cars and drivers in automated vehicles, the presented studies cannot provide solutions to address the interaction between a passenger and a manual driver. In general, there is much research on human-machine interface and assistance in traffic situations, but only a few approaches directly addressed discomfort. Nevertheless, some of the concepts used in the presented studies could be helpful to address passenger feelings and will be adapted in the following section.

## 3. Theory and passenger assistance concepts

**Figure 1:**
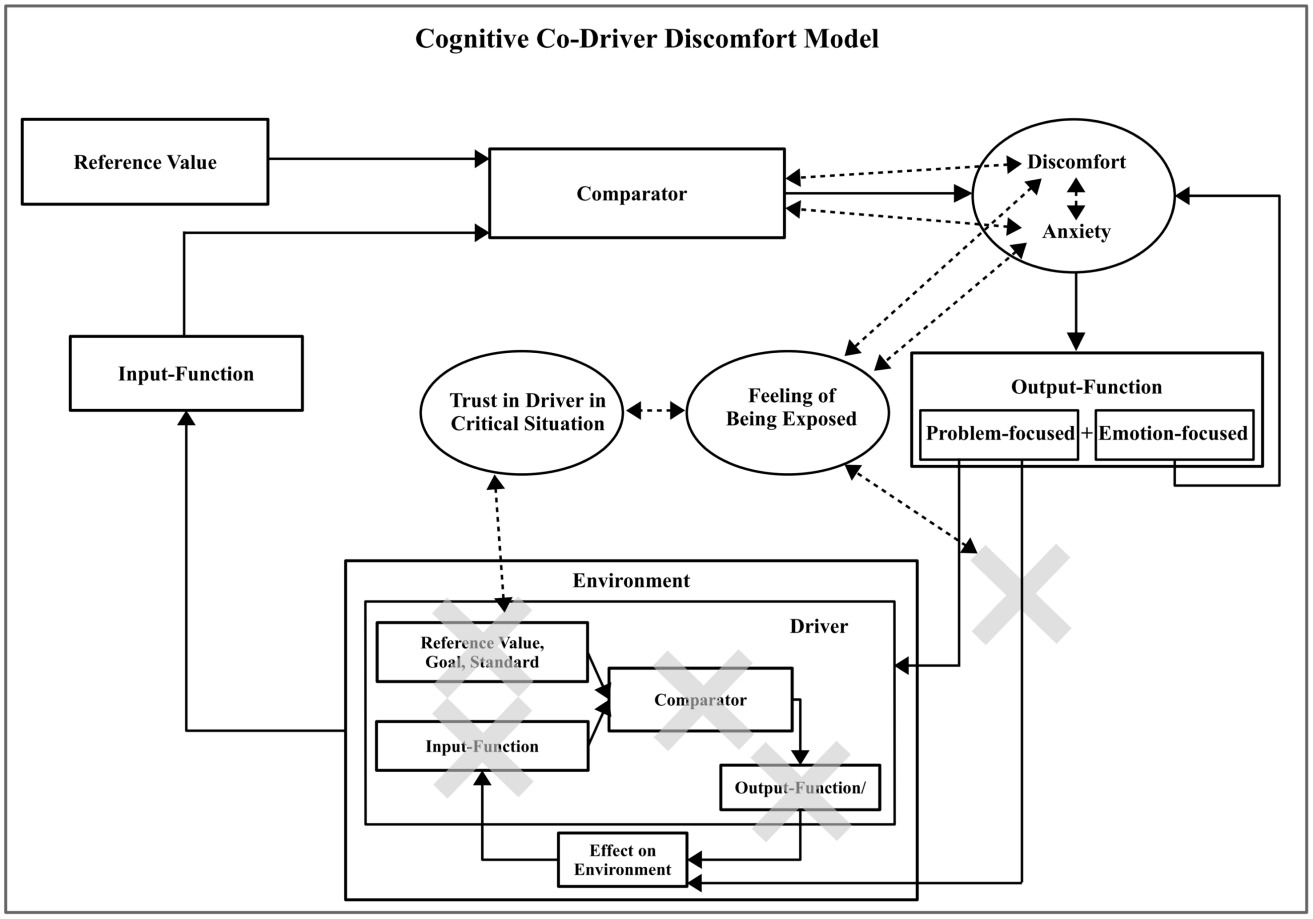
Cognitive passenger discomfort model based on Ittner et al. (2020). From the perspective of the passenger, the driver is part of the environment. Aspects in the model that are only sparsely or not available for passengers and therefore contribute to their discomfort are represented by gray crosses. The dashed arrows show correlated emotional concepts that were verified in the original study (Ittner et al., 2020).

For the development of passenger assistance systems, it is relevant to know which mechanisms influence discomfort in order to provide appropriate information in relevant situations. According to the cognitive passenger discomfort model introduced in Ittner et al. (2020) (adapted in Figure 1), the driver regulates the driving task in a feedback-loop (Miller et al., 1960; Carver and Scheier, 2002) with the environmental situation, where (s) he has full information about the own cognitive state at each point of the process (Figure 1 bottom, “Driver” box, without the crosses). The driver has information about his/her attention focus (Input-Function), his/her own driving experience and driving style preferences (Reference Value), about thresholds used for the estimation of a situation’s criticality (Comparator), and their planned actions (Output-Function). In contrast, a passenger has to consider the driver as part of the environment and receives no or only indirect information about the internal processes of the driver. This reduced or inaccessible information about the cognitive state of the driver is represented by the crosses in the “Environment” box in Figure 1. The lack of information can lead to a difference in the estimation of a situation’s criticality between driver and passenger. An overestimation of criticality by the passenger might lead to discomfort which can sometimes also be accompanied by anxiety. According to the cognitive passenger discomfort model, a lack of information can also negatively influence the trust in the driver in critical situations, influencing the passenger’s feeling of being exposed. This feeling of being exposed arises due to the passenger’s limited ability to intervene in such situations and contributes to their discomfort (represented by the rightmost cross in Figure 1). For a more detailed description of the model the reader is referred to Ittner et al. (2020).

Passenger discomfort can originate from (1) a lack of information about different aspects of the cognitive state of the driver (indicated by the crosses in the driver sub-model) or (2) a lack of control/influence, respectively problem-focused coping possibilities (indicated by the cross after the output-function of the passenger in Figure 1). These basic mechanisms are applicable in different situations and can be addressed through different assistance system concepts. The following general hypothesis will be investigated in this work:

*H1*: Provided information or means of influence by passenger assistance systems based on the cognitive passenger model leads to a reduction of passenger discomfort compared to a baseline without assistance

This work and the concepts in the model target situations where passenger discomfort is not justified through objective criticality. We, therefore, assume both baseline and assistance conditions to only contain situations that the driver can still control.

The relation between assistance system information and an increased trust in the driver and a better situation assessment, as implied by the model, are examined through correlations in order to validate the hypotheses:

*H1.1*: Displayed information or provided means of influence will impact the passenger’s trust in the driver, their criticality estimation of the experienced scenarios, and their discomfort

In order to investigate the influence of the individual mechanisms promoted by the model, sub-hypotheses will be formulated in the next paragraphs, and concepts for assistance systems used to address these mechanisms will be proposed. Each passenger assistance system will target one aspect that contributes to passenger discomfort.

### 3.1. Driver input-function

As the operator of the vehicle, the driver controls the vehicle based on the information (s) he receives (“driver input-function” in Figure 1). However, the passenger’s input-function does not need to match that of the driver, and (s) he therefore does not know if the driver is attentive and has seen all vehicles or traffic infrastructure that could become relevant for future driving. The uncertainty about the attentiveness of the driver can lead to an overestimation of a situation’s criticality and thus to discomfort. The passenger might feel exposed to the situation, which can be amplified if there is little trust in the driver. A related issue is addressed in many automated driving applications, for example, to improve take over times or take over performances (Hoff and Bashir, 2014), through sharing information with the driver about detected obstacles or hazards, which can be considered the system’s attention focus. Naujoks et al. (2017) visualized detected front vehicles with a human-machine interface. Another group used LED bars that light up to highlight detected hazards (Yang et al., 2018). In contrast to an automated vehicle, assessing a human’s focus of attention requires an additional inference step, for example through interpreting his/her eye movements. Visualizing the gaze targets or paths through a head-up display could provide information about the perceived environment details, similar to how it was used, albeit with a different target, by Trösterer et al. (2019) and Maurer et al. (2014). One possibility would be to highlight objects observed by the driver which could provide positive feedback about the attentiveness and reduce discomfort from uncertainties about detected obstacles. Alternatively, the system could analyze the gaze pattern of the driver with respect to objects deemed relevant and provide an abstracted signal of the results to the passenger. Using even higher means of abstraction, the system could measure the overall attention state of the driver, similar to conventional drowsiness detection systems (Ramzan et al., 2019), and communicate it through a passenger human-machine interface. The first human-machine interface concept will therefore target to investigate the following sub-hypothesis:

*H1.2*: Passenger discomfort is reduced by sharing information about the attentiveness of the driver compared to the baseline conditions without this information

### 3.2. Reference value

According to the cognitive passenger discomfort model, passengers might have no or limited information about the driver’s reference values, depending On familiarity with the driver. Reference values are used to ground the perceived input and are often formed through experience or habits (preferred driving style, driving experience, or accident history). In The context of The driving task, The reference value could Be The distance To The car In front that The driver Is used To, a driving speed that (s) He perceives As comfortable, or familiarity with certain aspects of an environment. There exist studies that showed that drivers who prefer smaller time headways (THW = distance to front vehicle/own velocity) Are often more skilled in braking control (Winsum and Heino, 1996). Transparency about these reference values could therefore be beneficial for the passenger’s trust in a driver. In line with the passenger discomfort model, this transparency could also lead to a more accurate estimation of situation criticality, which then could lead to lower passenger discomfort. Especially, if the passenger drives with an unknown driver like a taxi driver, this type of information could be useful to better estimate if a driver can be expected to deal with a situation. Addressing the related information asymmetry between a driver and an advanced driver assistance system, a study by Israel et al. (2010) presented a human-machine interface to provide the reference value of an adaptive cruise control system to the driver. The human-machine interface showed information about the actual distance to a vehicle in front together with a threshold distance marker. When the distance reached that threshold, the adaptive cruise control would start to brake. This type of information made it possible for the driver to better anticipate when the adaptive cruise control will start to brake (i.e., know the adaptive cruise control’s reference value) and when (s) he should intervene if not. A study by Khastgir et al. (2018) showed that information about an automated vehicle’s capabilities led to higher trust in the system, regardless of whether the capabilities or limitations were high or Low. A possibility for passenger assistance would be to provide information or statistics about a driver’s reference values or skills. Possible human-machine interface concepts could provide static information for example about The experience of a driver or more detailed information matching The current driving situation such As The average time headway from The driver’s past highway drives. For this concept the following sub-hypothesis will be evaluated:

*H1.3*: Passenger discomfort is reduced by information about reference values based on the driver’s experience compared to the baseline condition without this information.

### 3.3. Comparator

The cognitive passenger model implies another possibility that can influence the estimation of a situation’s criticality increasing passenger discomfort. The passenger does not know how the driver assesses different situations. For example, if (s) he incorrectly considers the distance to a front vehicle to be sufficient or if only the passenger incorrectly estimates it as too small. In terms of the model, this means that the passenger does not know how the driver evaluates the input in comparison to its reference value in the comparator. A possible solution would be to provide an objective technical system that takes over the role of the driver’s comparator, displaying to the driver and passenger, for example, the legal minimum distance. If this information would be available to both driver and passenger, the latter could rely on the fact that they are both using the same comparator for their evaluations. This leads to the following sub-hypothesis investigated for this assistance system:

*H1.4*: Passenger discomfort is reduced by explicit information about objective safety thresholds provided to both the passenger and the driver compared to the baseline condition without this information.

### 3.4. Driver output-function

Uncertainty regarding already executed actions or intentions of the driver is another potential cause of discomfort in the cognitive passenger model. This can be the case when a potentially critical situation is already taken care of as the driver is ready to brake, while this cannot be incorporated into the evaluation of a passenger as (s) he does not know the driver’s plans. This means that in certain situations, the passenger does not know about the driver’s braking intention. For the passenger, it is not always clear whether the driver, in preparation for a situation that requires braking, is only taking his/her foot off the gas or already has it on the brake. A similar effect is caused by the time delay between an applied braking force and the perception of deceleration by a passenger. With an assistance system displaying the actions or intentions of the driver, it would be easier for passengers to predict or more accurately evaluate the safety of a driver’s behavior. This transparency could lead to a more accurate estimation of the situation and to an increased trust in the driver. In a study by Löcken et al. (2016), information about the intentions of an automated vehicle improved the accuracy of the estimates of intentions and future maneuvers of the vehicle. Chang et al. (2019) visualized information about the intended path of the automated vehicle or of other road users. Participants trusted the automated vehicle more when having access to such a human-machine interface. Unfortunately, the early detection and visualization of human intentions is more difficult compared to the plans of an automated system. One approach to reduce perception delays could be to visualize the driver’s foot position with respect to the brake pedal. With this information, they could see that the driver is prepared to brake or did already start braking. Thus, the hypothesis under investigation for this concept is:

*H1.5*: Passenger discomfort is reduced by information about the braking intentions of the driver compared to the baseline condition without this information

### 3.5. Passenger output-function

When a passenger assesses a situation as more critical than the driver due to limited information (e.g., about the driver’s input−/output-function, comparator, or reference value), his/her ability to actively intervene or cope with situations in a problem-focused way is limited as they have no access to the vehicle controls. There is only an indirect possibility to influence the situation by asking the driver to change the driving behavior. However, the passenger has to rely on the driver to follow such a request. If this is not the case, the situation does not change, and discomfort stays high according to the passenger model, or might even increase by adding social discomfort. For that reason, some passengers might refrain from this coping option as the driver could understand this as a criticism of their driving style. Another way to cope with such situations is the passive emotion-focused way. This means that passengers could try to change their emotions for example to reduce discomfort through distraction. Interviews by Ittner et al. (2020) showed that a large proportion of passengers used emotion-focused strategies (42%) like controlled breathing, grabbing a door handle, or distracting themselves. In comparison, the problem-focused strategy of saying something to the driver or criticizing him/her was used less frequently (21%). Both strategy types showed mixed helpfulness. The limited possibility to cope with such situations can lead to a feeling of being exposed supporting passenger discomfort (Ittner et al., 2020). Providing additional means of control to passengers could therefore reduce the feeling of being exposed and consequently discomfort. Results of a study by Frison et al. (2017) support this conclusion. They showed that automated driving vehicles without any control satisfied driver needs significantly less than manual driving and that cooperative automation reduced the negative effects of pure automation. However, allowing a direct impact on driving maneuvers, as in cooperative driving approaches, might strongly interfere with the driving task in the manual driver-passenger context. There are studies (Hesse et al., 2013; Schieben et al., 2014) that show that automatically initiated steering interventions in conventional vehicles can avoid collisions, but drivers tend to counter steer or hold the steering wheel during these interferences. Some participants in a study by Schieben et al. (2014) explained that they find an automatic intervention frightening or feared a collision with traffic in the opposite lane. For passenger assistance, it might be a better alternative to provide additional means of indirect control. The passenger could, for example, have his/her own brake pedal, which does not control the vehicle but provides the driver with a signal indicating a braking suggestion. However, such communication with the driver might be more successful if it would be based on a situational context instead of an explicit action recommendation. The passenger could highlight a critical traffic participant to the driver using similar means as in Trösterer et al. (2019) or provide information about safety distances. In the context of this use case and the related aspect in the cognitive passenger model the sub-hypothesis is as follows:

*H1.6*: Passenger discomfort is reduced by a means to influence on the safety distance compared to the baseline condition without this means

## 4. Feasibility study – Passenger discomfort In simulation

To examine the impact of the proposed passenger assistance system concepts on passenger discomfort a simulator user study was set up. A simulation has the advantage that influences of the environment, like weather or traffic conditions, can be controlled, and therefore scenarios can be replicated for each participant. However, the results of such a user study can only be valid if it is possible to induce passenger discomfort in a simulator. Since prior research with passengers only used surveys or real driving studies (Ittner et al., 2020, 2021) and no simulator studies to investigate passenger discomfort, we decided to first test with a small feasibility study if passengers can experience discomfort in a simulator and if they can experience it without becoming motion sick. The effect of the assistance system concepts on passenger discomfort was then investigated in a subsequent extended simulator user study.

### 4.1. Methods

*N* = 9 participants (*n* = 5 male; *n* = 4 female) took part in the feasibility study. They were frequent passengers who were recruited from participants of the interview study reported in Ittner et al. (2020). When asked about the frequency of driving as a passenger, 22% reported being passengers 3–5 times per week, 56% reported 1–3 times per month, and the remaining 22% were passengers less than once per month. 55% of the participants reported being daily or almost daily drivers.

A static simulator with a full-body production car in a closed room facing five screens surrounding the vehicle was used. The simulation was done using the SILAB software^1^ (The figures in section 5.1.3. assistance systems show impressions of the visualization as seen from inside the vehicle). The frontal field of view of the simulator covers an angle of 300° (horizontal) and of 47° (vertical). The rendered scene is projected onto the screens using five projectors with a resolution of 1,400 × 1,050 pixels each. The side mirrors are replaced by LCD displays showing a simulated rearview. The conventional rearview mirror shows the virtual environment on another LCD in the trunk. The simulator was originally designed for participants on the driver seat, which implies specific tuning of the projection perspective. To avoid simulator sickness caused by the different viewing angles, the projection of the simulation was adjusted toward the passenger’s position.

The feasibility and the following user study were both approved by the institutional ethics committee at the WIVW GmbH. This ethics committee follows the recommendations of the Deutsche Forschungsgemeinschaft (2019). In both studies, written informed consent was obtained from each participant. The driver was an instructed expert, who was tested to not be susceptible to simulator sickness in this passenger-focused setting. To prevent an influence on driver trust for the passenger, the driver was treated like a participant in front of the passenger participants (i.e., it was explained that the driver will be interviewed by another experimenter in the preparation room after the experiment). The driving behavior for all scenarios was prerecorded so that no dangerous situations could occur and it was reproducible for every participant. During the experiments, the driver pretended to steer the vehicle but the steering wheel commands were not used as input to the simulator.

**Figure 2:**
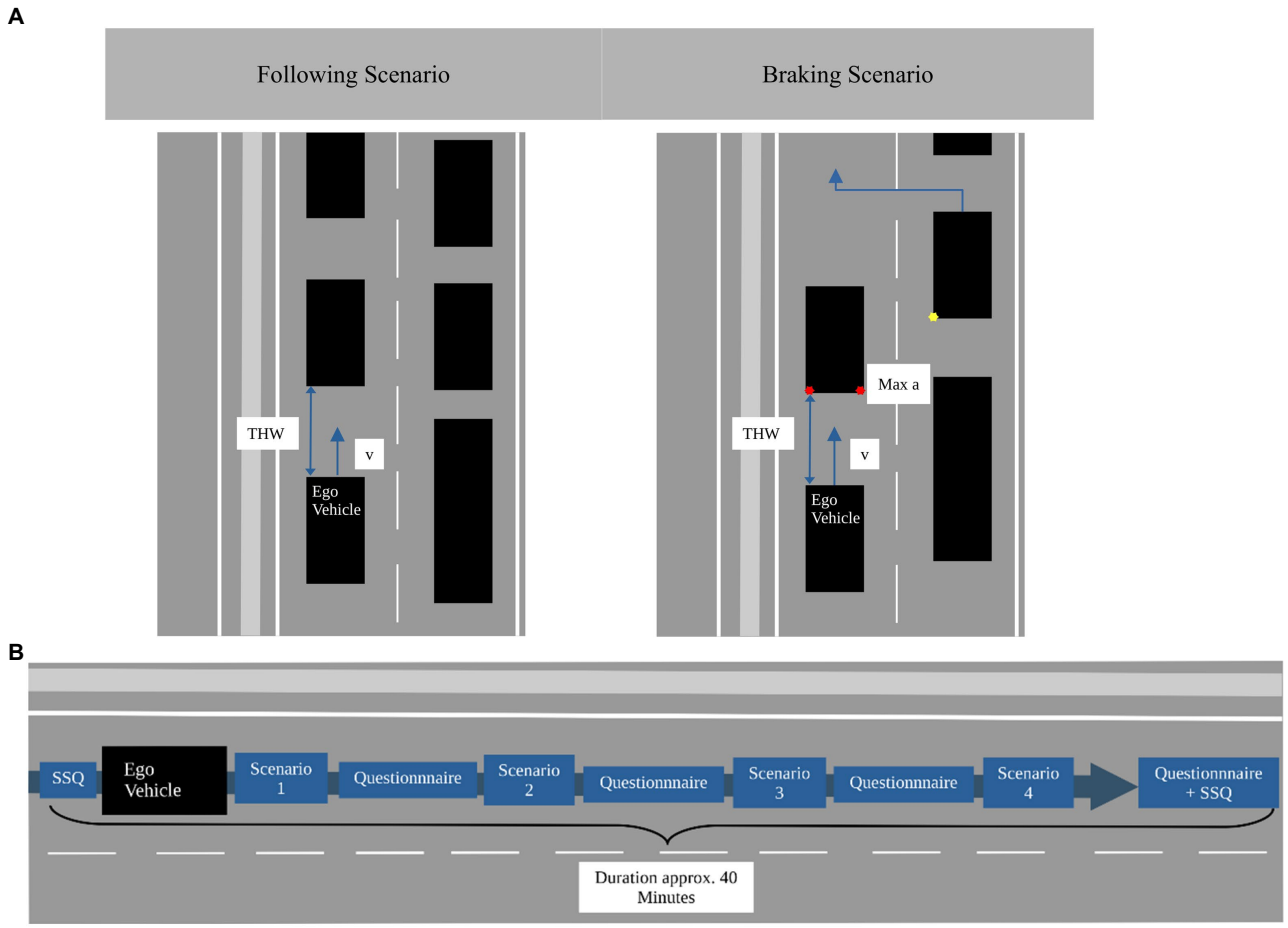
**(A)** Schematic flow of the following and braking scenario. **(B)** Procedure of a run in the feasibility study.

Results from previous interviews (Ittner et al., 2020) were considered for the design of the driving scenarios. Close following and high velocities were named as prominent reasons for passenger discomfort. Therefore, several ‘Following’ and ‘Braking’ scenarios were designed. All scenarios were experienced on a 2-lane highway, to investigate the effect of higher velocities on passenger discomfort, and with a constant, pre-defined time headway (THW). In the Following scenario (Figure 2A left), the car was driving behind several other cars on the left lane with *v* = 120 km/h while the right lane was crowded with vehicles and trucks so the driver was forced to stay on the left lane. The Braking scenario (Figure 2A right) was the same as the Following scenario except that a Truck changed its lane to the left and forced the cars in front of the ego-vehicle to brake strongly. Therefore, the ego-vehicle was likewise forced to brake. The two scenario types were experienced by the participants with both, a short (THW = 0.5 s), and a larger time headway (THW = 1.5 s; Note that statistics from real-world highway driving show that a significant number of drivers regularly drive at time headways between 0.5 and 1 s while the lowest setting for adaptive cruise controls is typically close to 1 s (Knospe et al., 2002; Ervin et al., 2005). In the Braking scenario, the front vehicle decelerated with a maximum of a = −4.5 m/s^2^ from approximately 120 km/h to 90 km/h. We selected relatively short time headways, as it is known, that situations appear less critical in a static simulator compared to those in real traffic due to a lack of motion cues, such as vestibular information, which are important for speed control or steering (Wierwille et al., 1983; Reymond et al., 2001). The 4 scenarios (2 Types * 2 THWs) were presented to the participants in a counterbalanced order in a single run. This run and the subsequent interview after each scenario took a total of about 40 min per participant. Figure 2B shows the procedure of a run. After each scenario, the experimenter interviewed the participants inside the car through an intercom. To measure the participants’ subjective discomfort, they were asked to rate it on a 16-point (0 = not at all…15 = very strong) category subdivision scale (Heller, 1985). Simulator Sickness was measured with the Simulator Sickness Questionnaire (SSQ) on a 4-point scale (none, slight, moderate, and severe intensity of simulator sickness symptoms; Kennedy et al., 1993a,b). To control for possible symptoms not induced by the simulator, participants completed the SSQ before and after the procedure. At the end of the study, the participants were informed about the fact that the driver was following an instructed driving style.

### 4.2. Results

Results show that most participants felt strongly uncomfortable during short time headways in the Braking scenario (*m* = 11.56, sd = 3.21), especially compared to scenarios with the longer time headway (*m* = 5.11, sd = 3.41). Detailed tests showed that this difference is significant (Asymptotic Wilcoxon-Test: *z* = −2.68, *p* < 0.05, *n* = 9, *η^2^* = 0.80). The same relation was found for the Following scenarios (Asymptotic Wilcoxon-Test: *z* = −2.53, *p* < 0.05, *n* = 9, *η^2^* = 0.71). In the Following scenario participants experienced medium discomfort during short time headways (*m* = 9.00, sd = 3.20) and low discomfort during long time headways (*m* = 5.89, sd = 3.37).

All participants completed the experiment without interruption of the procedure due to simulator sickness. The mean Total SSQ Scores before (*m* = 2.53, sd = 2.34) and after (*m* = 6.13, sd = 7.48) the procedure showed a slight increase of symptom severity, but the difference was not significant (Asymptotic Wilcoxon-Test: *z* = −1.52, n. s., *n* = 9). Only a few participants showed light symptoms after the procedure, one participant reported severe salivation and another one reported moderate eye strain after the last session. The simulator sickness specific symptom *Nausea* was also only reported to a small degree by a single participant. The driver showed no signs of simulator sickness during the whole study.

### 4.3. Discussion

The results show that participants can experience discomfort in a static simulator without experiencing simulator sickness on the front passenger seat if the field of view is adapted accordingly. The most effective settings were highway scenarios with close following of other vehicles, especially when the driver was suddenly forced to brake under these conditions. Although the discomfort ratings of the long time headway scenarios were significantly lower than the ratings of the short time headway scenarios, the mean values show that, with the exception of a few participants, passengers also experienced some discomfort in these scenarios. The same scenarios were also tested in a city environment (not reported here) which showed similar results regarding passenger discomfort. However, the following study will be restricted to the highway setting.

## 5. User study – Passenger assistance systems

The user study investigated the influence of the various assistance systems on passenger and compared these different concepts in this respect.

### 5.1. Methods

#### 5.1.1. Sample

The study in the static simulator was conducted with *N* = 40 participants (*n* = 21 female and *n* = 19 male). Again, they were frequent passengers who were recruited *via* the WIVW GmbH test panel. When asked about the frequency of driving as a passenger, 18% reported being passengers 3–5 times per week, 35% reported 1–2 times per week, 35% reported 1–3 times per month and the remaining 13% were passengers less than once per month. Efforts were made to ensure a similar distribution of participants in the categories of gender, age, passenger and driver experience across the subgroups (see Supplementary Table S4 in the supporting information). 65% of these participants reported being daily drivers. The age of the participants in the sample was between 21 and 68 years (*m* = 43.2 years, sd = 14.2 years).

#### 5.1.2. Scenarios

The scenarios used in this simulator study were similar to the highway scenarios in the feasibility study (Figure 2A) but with adapted values for time headway and deceleration. Based on the results of the feasibility study and the experience with static simulators, a focus was put on situations with a higher probability of causing discomfort for the participants. Therefore, it was decided to use only shorter time headways and increase the braking deceleration of the front vehicle. In the Braking scenario, the front vehicle decelerated with max a = − 12.5 m/s^2^ from approx. 120 km/h to approx. 70 km/h. One run consisted of six permutated scenarios, three Braking scenarios, and three Following scenarios with three time headways of THW = [0.3 s, 0.6 s, 0.9 s] each (scenarios and THW = within-subjects). At the beginning of each Following or Braking scenario, there was a part in which the driver approached some vehicles on his/her lane. After driving through one scenario, a short section without traffic followed in order to connect the scenarios.

#### 5.1.3. Assistance systems

In the following, five passenger assistance system designs based on the five concepts for reducing passenger discomfort introduced in section 3 and how they are used in the experiments will be described.

##### 5.1.3.1. Driver attention display

**Figure 3:**
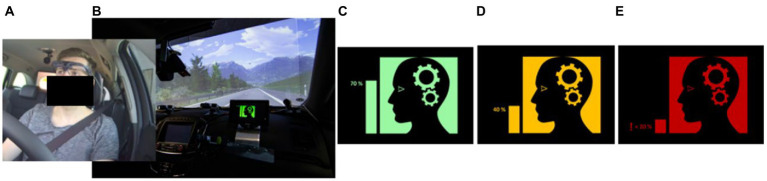
Driver attention display. **(A)** driver with eye-tracking glasses and two EEG electrodes in the temporal area. **(B)** Passenger display on the dashboard, showing the attention status of the driver. **(C–E)** Icons used for high (70–100%), medium (40%) and low/critical (<20%) attentiveness of the driver.

This assistance system variation aims to communicate that the attention of the driver is focused on the relevant traffic situation (Figure 3) based on the unknown driver-input aspect in the model. For the use case investigated in this work, this information should provide positive feedback about the attentiveness of the driver during distance regulation and his/her readiness for a reaction to sudden braking of the front vehicle. Participants were told that the attentiveness of the driver was measured *via* electroencephalography (EEG) in the temporal area and *via* eye-tracking (Figure 3A). It was explained that the data was used to determine how much the driver focuses on the road, that at different amounts of distraction the system state would change, and that glances to the mirrors were not treated as a distraction. Although the system functionality was hardcoded, we deliberately used more apparent measurement methods such as EEG or an eye-tracker to make it more convincing for the passenger that a functioning system evaluates the driver’s attention. The attention focus was visualized on a display with a color-coded icon and a bar plot providing an intuition for the “amount of attention” (Figures 3C–E). The mapping of the different colors, as explained to the participants, was green for high attentiveness (70–100%), yellow (40%) for a slight distraction of the driver, e.g., while changing the radio channel, and red for a critical and longer distraction of the driver (<20%), as it would be the case if the driver was reading a text message on a cell phone. To guarantee reproducibility, the driver’s attentiveness was not computed online but explicitly set based on the situation. As the main interest was to evaluate the system’s potential to reduce discomfort, the attention focus was constantly set to high, only providing positive information to the passenger. During the test scenarios, participants therefore exclusively saw the green icon (Figures 3B,C). The display showing this information was installed on the dashboard in front of the passenger (Figure 3B).

##### 5.1.3.2. Preferred time headway head-up display

**Figure 4:**
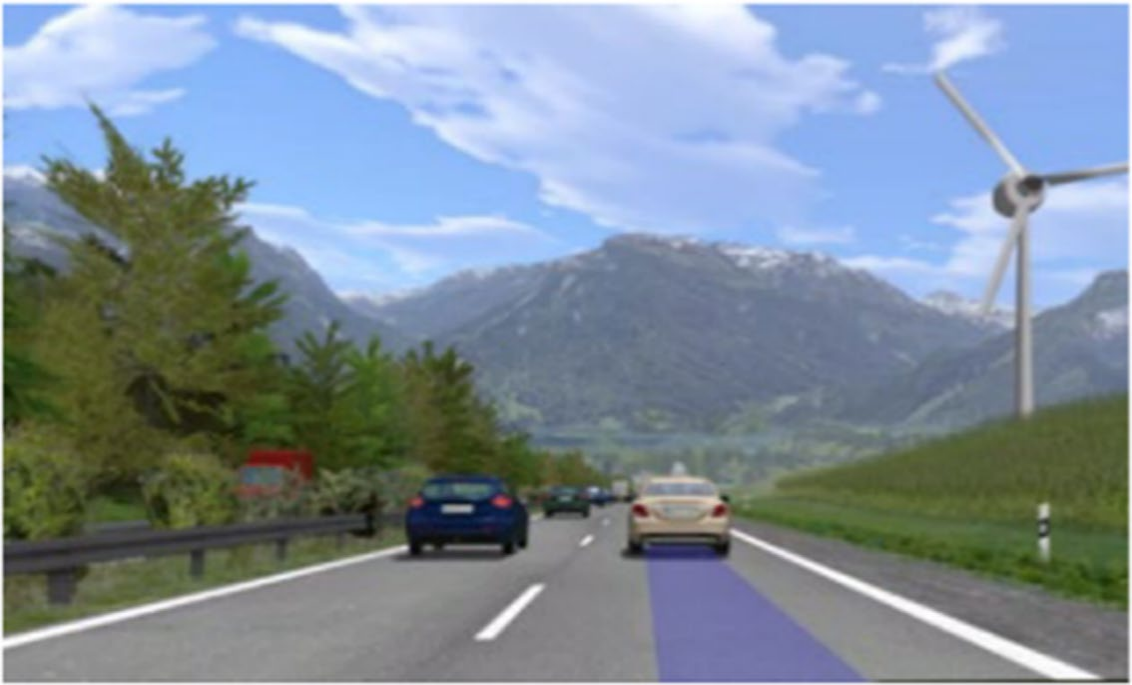
Preferred THW head-up display. Visualization showing a driver preferred time headway of 0.4 s and a front vehicle at the same distance.

This assistance system tries to communicate a reference value that a driver uses to evaluate following distances. This was approached by providing information through a simulated head-up display visible to both driver and passenger. It was rendered as a semi-transparent bar on the road between the ego-vehicle and the preceding car directly into the simulation environment (Figure 4). This type of visualization makes the information equally and intuitively available to both occupants. The semi-transparent bar had a blue color and a constant length. Participants were told that the length of the bar corresponds to the distance the driver is usually using. During the instruction ride, the driver was asked to drive with his/her personal preferred driving style and with a distance, at which they know they could still react in time to calibrate the system. However, as the driver was part of the experimental design, the actual preferred time headway used during the calibration was the same (THW = 0.4 s, which should represent a skilled driver within our scenario range) for all participants and in every test scenario. Additionally, at the start of an experiment, passengers in this condition got the information that the driver is experienced and never had an accident before. This aimed at creating the impression that the driver is able to react adequately even at small distances.

##### 5.1.3.3. Shared safety distance head-up display

**Figure 5:**
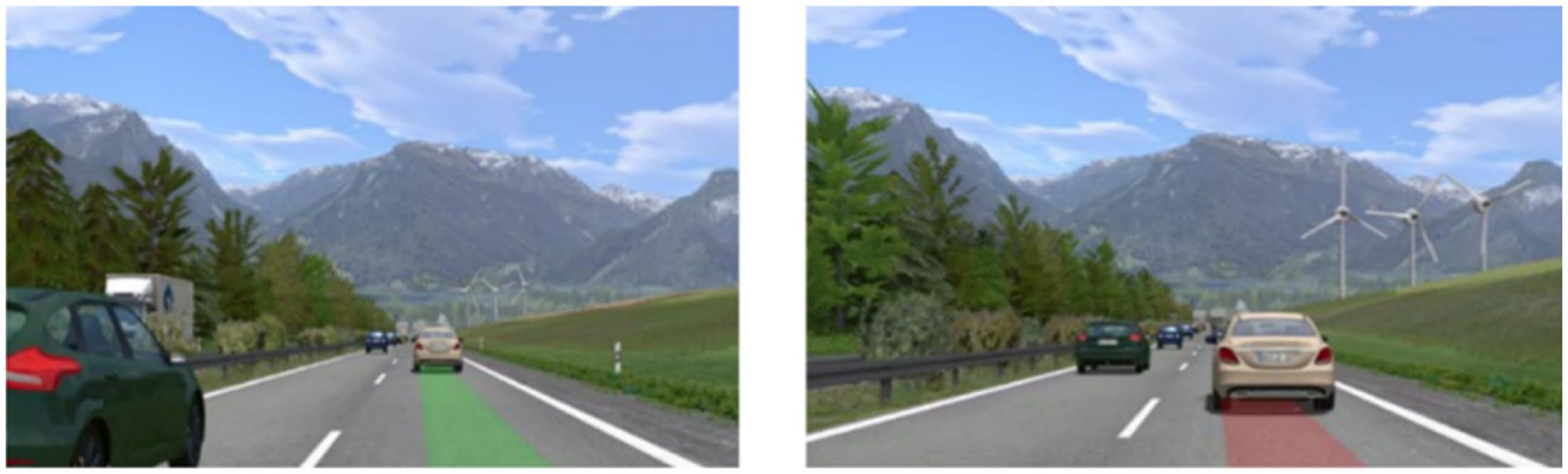
Shared safety distance head-up display. Left: Head-up display when the safety distance is sufficient. Right: Head-up display when the distance is below the safety threshold.

This passenger assistance system has the target to communicate to the passenger that (s) he has a shared understanding of a ‘safe’ distance to the front vehicle with the driver. This should provide information about the driver’s comparator function in the passenger model. The visualization was, similar to the “Preferred THW head-up display,” implemented as a semi-transparent bar on a head-up display, which had a green color as long as a defined safety distance was kept (Figure 5 left). When the distance was too small the bar changed its color to red (Figure 5 right). The participants received the explanation that the red color signals that the driver would no longer be able to react in time to a sudden break of the front vehicle due to physical constraints of the vehicle, and an accident could not be avoided. However, the safety threshold was set to 0.3 s so that participants always received positive feedback about the safety distance (green bar) to communicate all experienced situations as objectively safe.

##### 5.1.3.4. Braking information display

**Figure 6:**
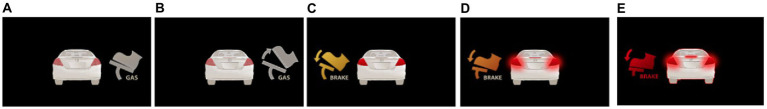
Braking information display. Icons shown when the driver has his foot on the gas pedal **(A)**, releases the gas pedal **(B)**, and applies different intensities of pressure to the brake pedal **(C–E)**.

This assistance system should display information about driver output relevant for the regulation of longitudinal velocity. The same display as used for the “At” system was used to show an abstracted information about the current position of the driver’s foot with respect to brake and gas pedal to the passenger (Figure 6). The human-machine interface used two Icons for positions on the gas pedal (grey): A foot on the gas pedal and a foot lifted from the gas pedal. For the brake pedal, three more icons were showing different braking intensities. Additionally, a vehicle icon was shown with three different brightness levels of the brake lights corresponding to the braking intensities. During the experiment, the display represented the gas and brake pedal pressure as used for the input in the simulation software. If any gas pedal pressure was registered, the first icon (Figure 6A) was shown, the next was shown when no pedal was pressed and the symbols (C)-(E) represented <30%, 30–70%, and > 70% of the brake pedal pressed.

##### 5.1.3.5. Active distance influence head-up display (Button/Bu)

**Figure 7:**
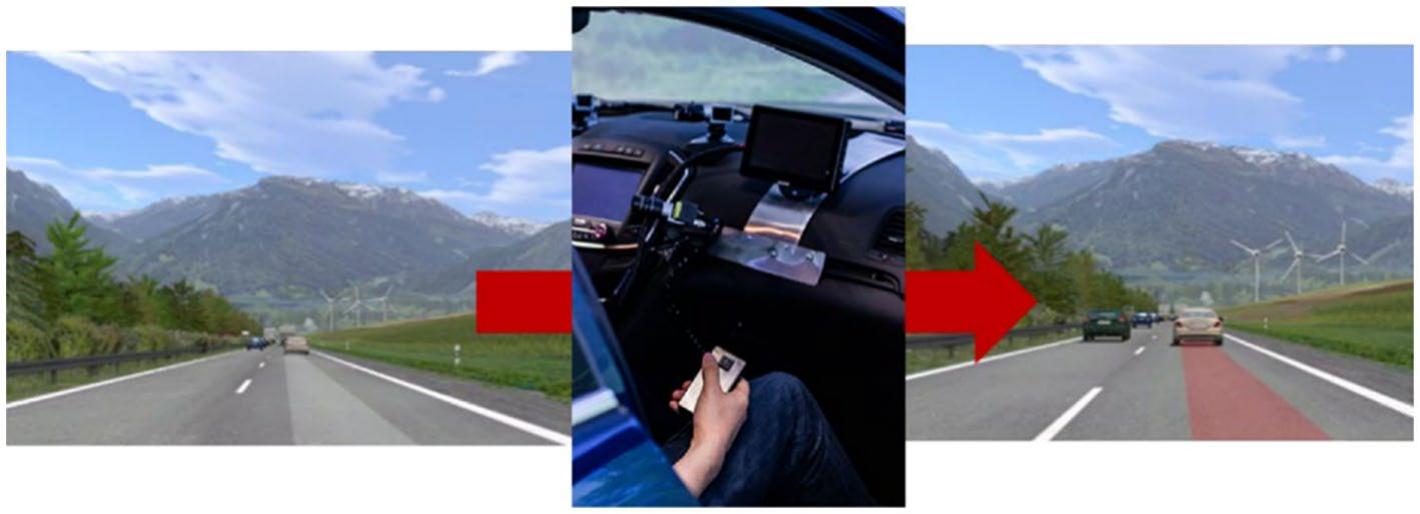
Active distance influence head-up display. Left: Head-up display when the passenger does not intervene. Middle: Passenger intervention using a handheld controller. Right: Head-up display after the passenger pressed the button, independent of actual distance.

The visualization of this system is similar to the shared safety distance system. The functional difference is that the passenger could decide when the color of the bar would change from gray to red (and back) using a hidden button (Figure 7) attached to the right side of the front passenger seat. With this, they could signal the driver whenever they think that the safety distance felt too small. By pressing the button again, the passenger could change the color from red to gray to show the driver that the distance is sufficient again. This offers the passenger the possibility for a new problem-focused coping strategy (passenger output-function), but compared to direct communication, provides a situation-embedded and unemotional channel. The participants got the instruction that the driver was not informed that they have such a controller, but that the driver got the same information as for the shared safety distance system. This should communicate, that the driver would receive safety distance information based on an objective measure.

#### 5.1.4. Procedure

**Figure 8:**
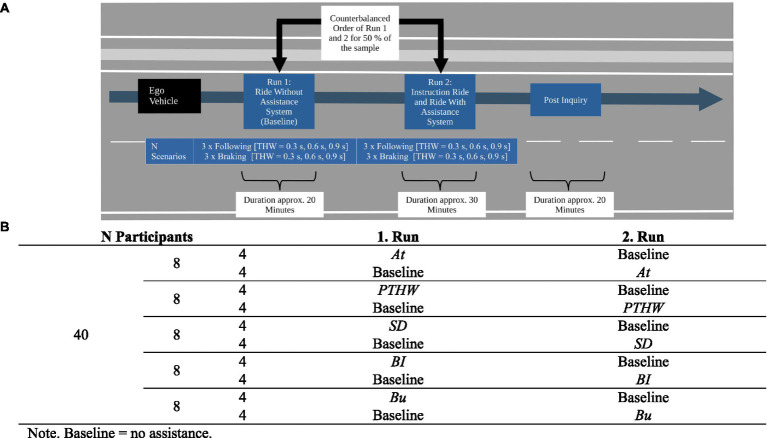
**(A)** Procedure of the passenger assistance system simulator study. **(B)** Study design with an overview of the distribution of the subjects.

The study was conducted in the same simulator as the feasibility study. The duration of the experiment was 1.5 h per participant, in which they experienced two runs of each 20 min

as a passenger on a two-lane highway. The remaining time of 50 min was used for the introduction of the assistance systems and interviews after the session. Every participant experienced one run without assistance and one run with one of the passenger assistance systems (system = between-subjects with a sample of *N* = 8 participants per assistance system). The order of the two runs was counterbalanced (Figure 8A). Before the run with assistance, the driver and each passenger experienced the range of information that the respective human-machine interface can display in a suitable situation with an explanation by the experimenter. During the road sections, which connected the scenarios, the passengers answered the scenario-specific questions. The questions were organized in a folder that allowed them to answer the questions covertly in front of the driver. This should prevent the participants from giving lenient ratings in the scenarios because the driver could see a negative evaluation and take it as criticism. For an overview of the study design, see Figure 8B.

As in the feasibility study, the drivers were two instructed experts, which was not revealed to the participants at the start of the study. The drivers drove with an instructed driving style (e. g. how to behave and react in the different scenarios, including to follow the feedback of the assistance systems at all times) and a simulated adaptive cruise control function was used to ensure a consistent driving style in terms of acceleration, braking and speed variations for all participants. In presence of the participants, the drivers were told that it is their own decision whether to react to the system’s feedback or not. This method was used to guarantee the greatest possible standardization and comparability of the driving style for each participant while allowing for uncertainty about the driver’s reactions. At the end of the study, participants were informed about the fact that the driver followed an instructed driving style.

#### 5.1.5. Dependent variables

**Figure 9:**
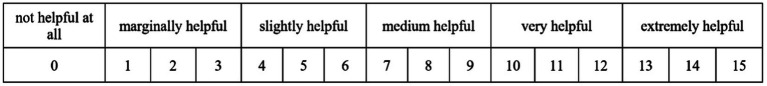
16-point category subdivision scale (Heller, 1985) used for the rating of system helpfulness after each scenario and in the post inquiry.

As in the feasibility study, after each scenario, subjective discomfort was rated on a 16-point category subdivision scale (Heller, 1985; 0 = not at all…15 = very strong). In the same way, participants were asked to estimate how safety critical the scenario was, their trust in the driver and how much they felt exposed to the scenario (see Supplementary Table S1 in the supporting information for complete formulations of the items). After each scenario with assistance system, the participants also rated how helpful the presented information/the influence means was in general and how helpful the assistance system was to better assess the scenario (Figure 9).

In the post inquiry, participants rated how helpful the assistance system was to reduce their discomfort in general with the same 16-point category subdivision scale, and they were asked if they would like to use the experienced passenger assistance system in a future car.

#### 5.1.6. Statistical methods

The statistical analysis was executed with the software package IBM SPSS 25. We chose to use non-parametric dependent asymptotic Wilcoxon tests (one-tailed) for our statistical analyzes because of the smaller sample size when testing hypotheses 1.2–1.6. For the investigation of the general hypothesis 1 and the relationships between the variables mentioned in hypothesis 1.1, parametric tests were used because of a sufficient sample size.

### 5.2. Results

#### 5.2.1. Scenario ratings

**Figure 10:**
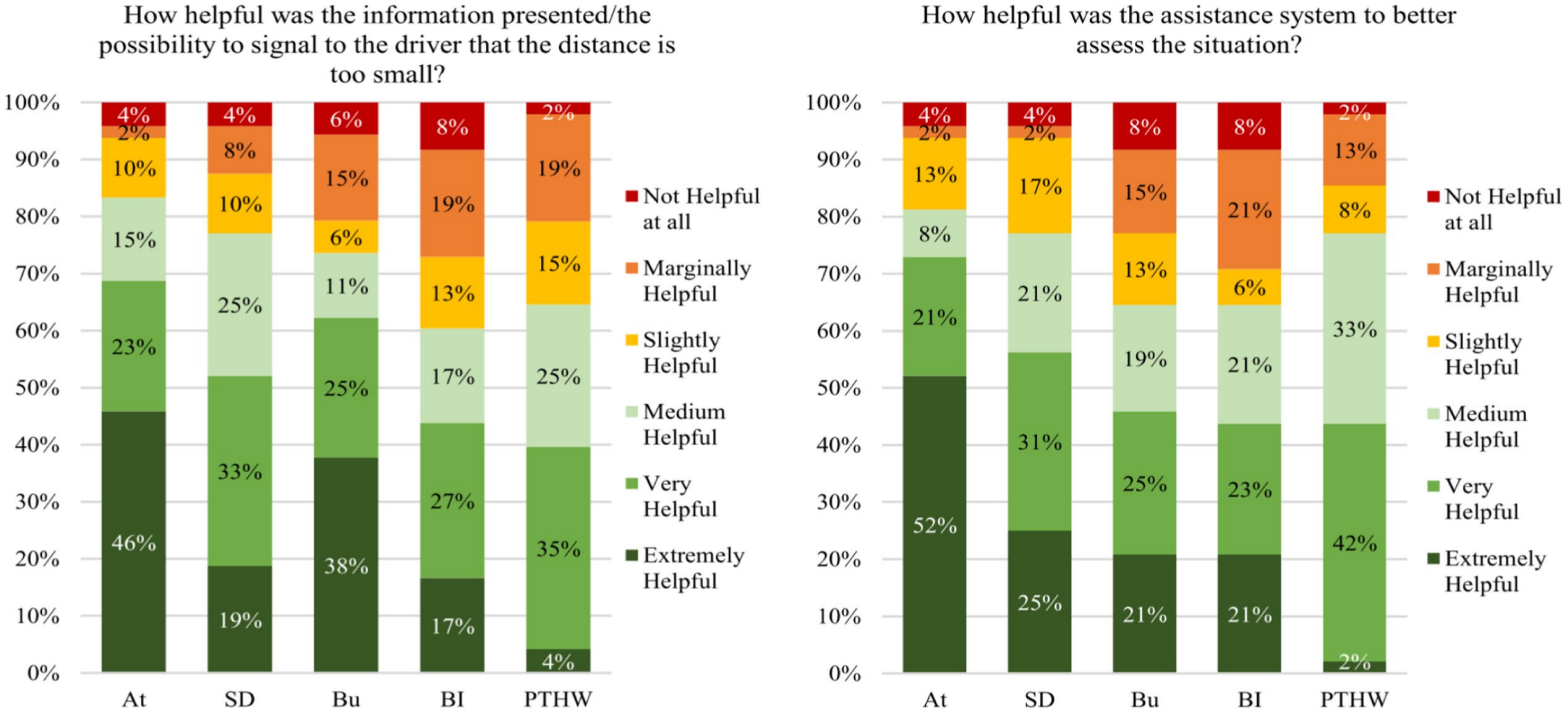
Distribution of scenario specific ratings regarding (left) the helpfulness of the present information, respectively, the possibility to have influence, and (right) the helpfulness of the assistance system to better assess the scenarios (right).

Figure 10 (left) shows the distributions of the information/influence helpfulness ratings over all 48 scenarios driven by all *N* = 8 participants (6 scenarios per participant) for each assistance system. The participants rated information presented by the “Attention” passenger assistance system as very or extremely helpful in 69% of the scenarios and in an additional 15% of the scenarios, the presented information of the system was rated as medium helpful. Similarly, in 60% of the scenarios, the participants rated the information and influence possibility provided by the “Button” system as very or extremely helpful, and in 13% of the scenarios as medium helpful. For the “Safety Distance” assistance system, the proportion of scenarios in which the information was rated as very or extremely helpful was slightly lower with 52%. On the other hand, the proportion of scenarios in which the information was rated as moderately helpful was higher with 25%. The information presented by the other two assistance systems was very or extremely helpful in less than 50%. The distribution of ratings for the question of how the assistance systems were helpful to better assess the scenario was similar (Figure 10 right). The “At” passenger assistance system was again very or extremely helpful in most of the scenarios (73%) followed by the “SD” (56%) and “Bu” (46%) systems. The other two assistance systems were very or extremely helpful in fewer scenarios (44% for “BI” and “PTHW” each).

Correlations were used to investigate the influence of the information or control provided by the assistance systems on participants’ discomfort or trust in the scenarios. Table 1 shows for each assistance system the correlations between the helpfulness of the information/the possibility to have influence and the perceived criticality of the scenario, the experienced discomfort, the trust in the driver, and the helpfulness of the assistance system to better assess the scenario. There are significant relations between the helpfulness of the information displayed by the “At” passenger assistance system and all other variables. The more helpful the information was rated, the less critical the scenarios were estimated and the less uncomfortable the participants felt. They also trusted the driver more and rated the assistance system as more helpful to better assess the scenario. The relation between the helpfulness of the information provided by the assistance systems and the helpfulness of the systems to better assess the scenario was found for all variations. There were also significant connections for the “Bu” system’s helpfulness to the variables “Criticality,” “Discomfort,” and “Trust in the driver.” However, the direction of this relation was contrary to the connection found for the attention assistance system. The more helpful the participants rated the possibility to signal to the driver that the time headway is too small, the more critical and uncomfortable the scenarios were assessed. Additionally, the more the trust in the driver was reduced, the more helpful the possibility to intervene in the scenarios was rated. The other assistance system variations showed no relations to the other variables.

**Table 1:** Pearson correlations between the helpfulness of the displayed information/provided influence and the variables criticality, discomfort, trust in the driver and the helpfulness of the assistance system to better estimate the scenario by assistance system.

System (*N* = 48)	Variables	Info/Influence Helpfulness (Pearson)	*p*
At	Criticality	*r* = −0.63	<0.01
	Discomfort	*r* = −0.65	<0.01
	Trust	*r* = 0.70	<0.01
	Estimate	*r* = 0.97	<0.01
SD	Criticality	*r* = 0.17	n.s.
	Discomfort	*r* = 0.10	n.s.
	Trust	*r* = −0.13	n.s.
	Estimate	*r* = 0.96	<0.01
Bu	Criticality	*r* = 0.41	<0.01
	Discomfort	*r* = 0.50	<0.01
	Trust	*r* = −0.43	<0.01
	Estimate	*r* = 0.89	<0.01
BI	Criticality	*r* = 0.13	n.s.
	Discomfort	*r* = 0.04	n.s.
	Trust	*r* = 0.12	n.s.
	Estimate	*r* = 0.98	<0.01
PTHW	Criticality	*r* = −0.11	n.s.
	Discomfort	*r* = −0.08	n.s.
	Trust	*r* = 0.16	n.s.
	Estimate	*r* = 0.87	<0.01

n.s., not significant. *p* > = 0.05.

The *N* = 40 participants experienced *N* = 6 scenarios per person resulting in *N* = 240 scenarios with an assistance system and *N* = 239 scenarios without an assistance system because one participant had forgotten to rate in one scenario. In a first step, the main effect of reducing discomfort was investigated for all assistance systems together. In total, participants experienced a reduction of discomfort in comparison to the same scenarios without an assistance system in *N* = 116 (49%) cases. In *N* = 71 (30%) scenarios there was no change in the discomfort rating and in *N* = 52 scenarios (22%) there was an increase in discomfort. In the following, subgroup comparisons on the different levels are examined in terms of discomfort reduction of the assistance systems. Since we tested the different levels on the same data, a Bonferroni-Holm alpha adjustment was made. All tests that are significant after a Bonferroni-Holm alpha adjustment are marked with a “†” in the following. Paired *t*-Tests (one-tailed) showed a significant reduction of discomfort by the assistance systems (*t* = −4.63, *p* < 0.001^†^, *n* = 239, *d* = −0.300). This was found for assistance systems providing only information (Paired t-Tests (one-tailed): *t* = −3.50, *p* < 0.01^†^, *n* = 191, *d* = −0.253) and for the assistance system which provided control (Paired *t*-Tests (one-tailed): *t* = −3.38, *p* < 0.01^†^, *n* = 48, *d* = −0.487).

**Figure 11:**
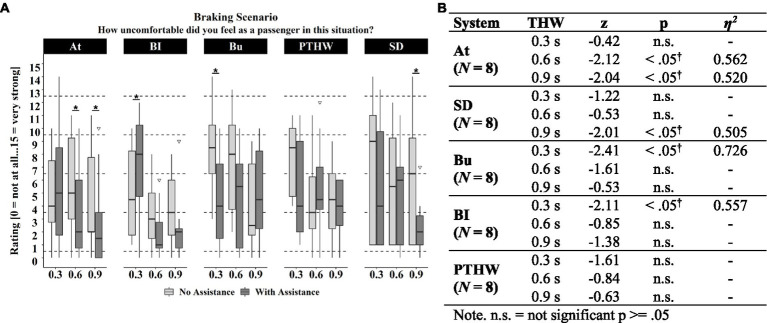
**(A)** Discomfort ratings experienced during the Braking scenarios for each time headway with and without assistance system across the different systems. Significant (*p* < 0.05) differences between ratings with and without assistance system are marked with *. Box range = Q1 to Q 3. Whiskers = 1.5 * IQR. **(B)** Asymptotic Wilcoxon-Tests for discomfort differences during Braking scenarios between assisted and baseline rides by time headway and assistance system.

In the next step, detailed tests were used to investigate the effects of each assistance system in the Braking and Following scenarios during different time headways. Figure 11A shows for each system the discomfort ratings by the participants during the Braking scenarios. For short time headways, a significant reduction of discomfort was only found for the “Bu” system, while for medium and long time headways there was a significant reduction by the “At” and the “SD” systems (Figure 11B). The “BI” assistance system seemed to even increase discomfort at short time headways. The system “Preferred THW” showed no significant reduction of discomfort for any setting.

**Figure 12:**
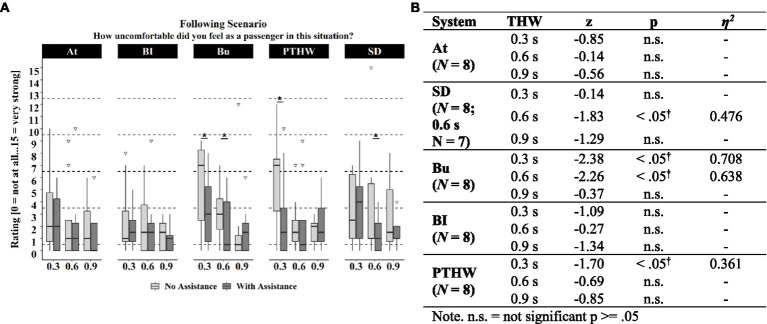
**(A)** Discomfort ratings experienced during the Following scenarios for each time headway with assistance system and without across assistance systems. Significant (*p* < 0.05) differences between ratings with and without assistance system are marked with *. Box range = Q1 to Q 3. Whiskers = 1.5 * IQR. **(B)** Asymptotic Wilcoxon-Tests for discomfort differences during following scenarios between assisted and baseline rides by time headway and assistance system.

Figure 12A plots for each assistance system the experienced discomfort for the Following scenarios. The “Bu” system reduced discomfort during short and medium time headways, the “PTHW” system during short ones and the “SD” system during medium ones (Figure 12B). For the other systems no significant effect was found.

Comparing the average discomfort ratings for the Braking and Following scenario without an assistance system, it appears that the participants experienced more discomfort during the Braking scenarios than during the Following scenarios. The average discomfort ratings of the baseline in the Following scenarios were generally at a very low level, showing that there was little discomfort to be reduced by a passenger assistance system.

When the “Bu” system was available, participants felt significantly less exposed during short and medium distances in the Braking scenarios compared to runs without an assistance system (Table 2). There was no reduction in their feeling of being exposed during the Following scenarios nor during Braking scenarios with larger time headways.

**Table 2:** Asymptotic Wilcoxon-Tests for differences of feeling exposed between “Bu” assisted and baseline rides by time headway and scenario.

Scenario (*N* = 8)	THW	*z*	*p*	*η^2^*
Following	0.3 s	−1.61	n.s.	-
	0.6 s	−1.69	n.s.	-
	0.9 s	−0.94	n.s.	-
Braking	0.3 s	−2.37	<0.05	0.702
	0.6 s	−2.03	<0.05	0.515
	0.9 s	−0.11	n.s.	-

n.s., not significant. *p* > = 0.05.

#### 5.2.2. Post inquiry

**Figure 13:**
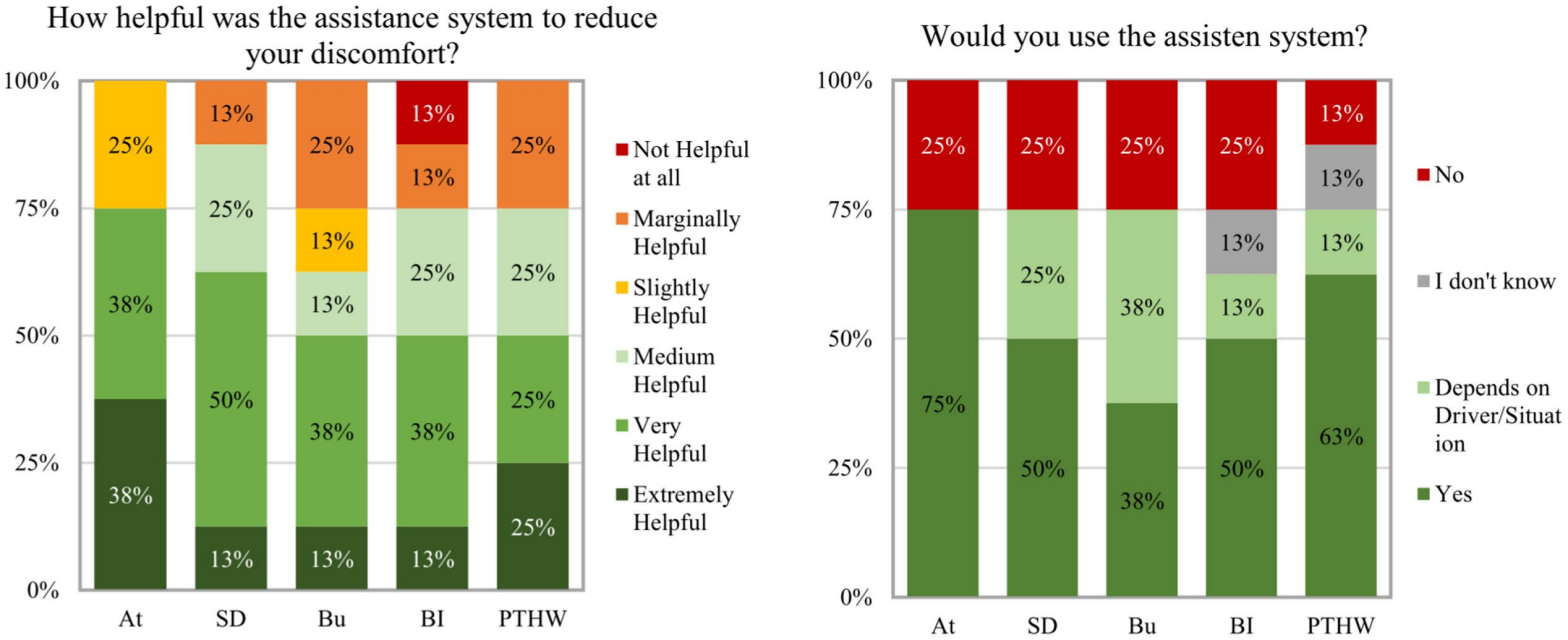
Left: Helpfulness ratings for the different assistance systems made in the post inquiry. Right: Reported intention to use the experienced assistance system.

The overall ratings in the post inquiry show that 75% of the *N* = 8 participants (Figure 13 left) found the “At” passenger assistance system very or extremely helpful in reducing their discomfort. Most of them argued that they felt more secure or had a positive feeling when knowing that a driver was focused. The “safety distance” system received positive responses from 62.5% of the participants, who often reported that they could better assess the distance or scenario. Additionally, 25% rated this system as medium helpful. 50% of the participants who experienced the “Bu,” the “BI” or the “PTHW” passenger assistance system rated it as very or extremely helpful in reducing their discomfort. The proportion of participants who reported the respective assistance system as slightly or marginally helpful was highest for the “Bu” system with 38%. Most participants stated that the system has no effect or could increase the anxiety of a passenger. There was only one participant who found a passenger assistance system (“BI”) not helpful at all in reducing discomfort with the argument that information about the driving or braking process would not be relevant to a passenger. For more participant responses to this subjective question, see Supplementary Table S2 in the supporting material.

The distribution of responses to the question of whether the participants would use the experienced assistance system was similar (Figure 13 right). The proportion of participants who answered that they would use the assistance system was highest for the “At” and the “PTHW” system with 75 and 62.5% followed by the systems “SD” and “BI.” Additionally, 25% in the “SD” group said that it depends on the driver or scenario whether they would use the system. In the “BI” group this amount was slightly lower with 12.5% because the other 12.5% of the participants were undecided. The lowest number of positive responses was found in the “Bu” system group with 37.5% who would use the assistance system. 37.5% in this group said that it depends on the driver/situation whether they would use the system. The remaining 25% would not use the system because they did not see a need for it. They reported that instead, they would say something to the driver (*N* = 2). Additional responses to this question can be found in Supplementary Table S3 in the supporting information.

### 5.3. Discussion

This section will discuss the results of the user study with respect to the hypotheses formulated in section 2. The general hypothesis 1 was that information about the cognitive state of the driver or a means for control can reduce passenger discomfort compared to rides with no information or control. Increased transparency should lead to an improved estimation of a situation or to the validation of such estimates which would reduce or prevent passenger discomfort. Providing means of control for the passenger should reduce their feeling of being exposed which consequently would reduce their experienced discomfort. The main effect of the information-specific assistance systems and the control-specific assistance system showed a significant reduction in passenger discomfort which confirms the main hypothesis.

Hypothesis H1.2 investigated if information about the attentiveness of the driver reduced passenger discomfort in comparison to a baseline without such information. Results showed that hypothesis 1.2 addressed through the “At” passenger assistance system can be accepted. The “At” system provided positive feedback about the attentiveness of the driver and implied the driver’s ability for a reaction to sudden braking. The information about the attentiveness of the driver significantly reduced the experienced discomfort in the simulator study. However, this was only found for medium and large distances during the Braking scenario. It is possible that the short distances were too small for the participants to trust the system. This is supported by the significant relation between the helpfulness of the information displayed by the assistance system and the discomfort and criticality ratings in the scenarios. The lower the participants rated the helpfulness of the assistance system in the experienced scenarios, the lower was their trust in the driver and the higher was the rated criticality and their experienced discomfort (H1.1). In the Following scenarios, the system showed no reduction. This could be due to the fact that baseline discomfort for all time headways was already very low. These very low discomfort ratings imply that the participants did not need an assistance system in these scenarios. Interestingly, in the feasibility study, discomfort ratings for the Following scenarios were much higher even though the time headways were larger. This could be caused by higher acceleration rates in the feasibility study which could have led to the impression of a more “aggressive” driving style.

Hypothesis H1.3 was concerned with the potential discomfort reducing effect of knowing the driver’s experience-based reference value. The assistance system “PTHW” communicated this reference value to the passenger for a more accurate estimation of a situation. The results for the “PTHW” assistance system were similar to the “BI” assistance system. The system only reduced passenger discomfort during short time headways in the Following scenarios. Hypothesis 1.3, therefore, has to be rejected. These results do not correspond to the findings by Khastgir et al. (2018) which showed that information about system capabilities increased driver trust, regardless of whether the capabilities were high or low. One reason named by the participants in the post inquiry was the subjective character of the “PTHW,” which means that it does not automatically correspond to a safe distance. The references used by an automated driving system as in (Khastgir et al., 2018) might instead be considered more objective. Based on the design, the relevance of the information provided by the “PTHW” depends on the familiarity of the driver. It is likely more informative when the driver is unfamiliar to the passenger like a taxi or lift driver. Although this was the case in the experiments, it did not seem to produce the desired anchor. This might be related to the very short preferred time headways that were used to cover the different scenarios.

Hypothesis H1.4 examined the discomfort reducing effect of explicit information about the safety threshold provided to the passenger and driver. The “SD” passenger assistance system aimed at communicating that both, the driver and the passenger, have a shared understanding of a “safe” distance to the front vehicle. The helpfulness of the “SD” information correlated positively with situation understanding. However, there was no relationship to trust or criticality estimation (H1.1). The assistance system only showed a discomfort reduction during long time headways in the Braking scenarios and during medium time headways in the Following scenarios. This means that the positive effect of transparent information indicated by the results of the study by Chang et al. (2019), was only partly found for this system variation. However, in the post inquiry more than half of the participants rated the assistance system as very or extremely helpful in reducing their discomfort. This leads to the conclusion that hypothesis 1.4 can be only partially accepted.

In hypothesis H1.5 it was investigated if information about the braking intentions of the driver could reduce passenger discomfort. Based on the results of the corresponding “BI” passenger assistance system, hypothesis 1.5 must be rejected. The system displayed information about driver actions that are relevant for the regulation of longitudinal velocity. This aimed to reduce uncertainties regarding already executed actions or intentions of the driver. The results showed no reduction of passenger discomfort in all scenarios and even increased discomfort during short time headways in the Braking scenarios. There was also no positive effect of braking information on trust in the driver. One possible explanation could be that the displayed braking process of the driver was perceived as a warning or a highlighting of criticality in the scenarios, increasing discomfort. Another explanation could be that the braking information was displayed too late to have the same positive effect as the more predictive information about the intentions of an automated vehicle like in the study by Löcken et al. (2016) or Chang et al. (2019). The information displayed by the system was also rated as helpful in fewer scenarios compared to the other systems.

The last hypothesis H1.6 examined if passenger discomfort is reduced by having influence on the safety distance. The “Bu” system provided means of indirect control of the distance to a front vehicle and through this an additional way for the passenger to cope with the situation. In contrast to the other systems, the “Bu” system also showed a reduction during short distances and in the Following scenarios. In some of the Braking scenarios, the system also led to a reduction in the participant’s feeling of being exposed. This is also in line with the positive influence of control during automated driving found by Frison et al. (2017). Therefore, hypothesis 1.6 can be accepted. Despite a more positive effect on passenger discomfort than other passenger assistance system concepts, it was rated as less helpful in the post inquiry compared to the “At” system and showed the lowest number of participants who would use the system. This could be explained by the fact that some participants argued that they do not need such a system because they would say something to the driver or would prefer an automatic system. For the “Bu” system the relations showed that the higher the trust in the driver was rated, the lower the helpfulness of the provided control and the discomfort was (H1.1). This indicates that the passengers did not need the assistance system in scenarios in which they trusted the driver to handle it. The positive relation between helpfulness and discomfort as well as the negative relation between helpfulness and trust in the driver implies this. The more helpful the possibility to signal something to the driver (need for control) was rated, the higher the discomfort and the lower the trust in the driver was in these scenarios.

All in all, the presented information about the cognitive state of the driver seems mostly helpful for passengers during medium time headways. Information-focused passenger assistance system concepts aimed to help passengers to verify their estimations and prevent discomfort, while the button system was designed to create a possibility to intervene when a situation already made passengers feel uncomfortable, which is especially the case during the short time headways. Since the smallest time headways used were very short, it can be expected that even with the changed distance perception in a simulator environment also many drivers would rate this critical and it could therefore be appropriate for passengers to feel uncomfortable in these situations. Following this line of arguments, it could be expected that participants did not calm down when receiving additional information about the situation but only considered an intervention helpful. The scenario ratings and the post inquiry ratings showed differences in the evaluation of the assistance systems. Except for the “Bu” system, the systems were rated more helpful in reducing discomfort in the post inquiry than their discomfort reducing effect in the scenarios implied. This could be caused by the low discomfort ratings in the following scenarios even without an assistance system which reduced the number of scenarios in which the participants needed information. But for the scenarios in which they felt discomfort, the systems were experienced as helpful.

As mentioned in the introduction of the feasibility study, simulator studies, as well as real driving studies, have their advantages and disadvantages. Besides the higher safety and controllability in simulator studies, they have the disadvantage of being less realistic. The simulator used in this study was a static simulator without available longitudinal forces, which likely reduced the perceived criticality of the scenarios. The aim of future studies could be therefore to examine the influence of the tested passenger assistance systems on discomfort under real driving conditions.

### 5.4. Limitations and future research

In the following paragraphs, possible limitations of the study are discussed and further points for future questions are considered.

The different passenger assistance system concepts presented in this work were mainly developed for the two most frequently reported reasons for passenger discomfort (Ittner et al., 2020): close following and fast driving. This means that except for the general “At” system, whose information could also be helpful during other situations, the systems presented information would mostly be relevant during close following on the highway. However, it is also possible to apply the cognitive passenger discomfort model to other situations in which the driver regulates the driving task such as overtaking maneuvers or complex city traffic. Therefore, it would be possible to derive other human-machine interface variations based on the passenger discomfort model presented in section 3 providing different types of information about the cognitive state of the driver or providing other forms of influence. Investigating what influence other types of information may have on passenger discomfort could be part of future studies.

Many of these aspects become particularly relevant when explicitly designing the exact interfaces for one of the passenger assistance system concepts. The designs used in this study were chosen to clearly relate to the concepts but without proposing or evaluating them with respect to effectivity or side effects. It is, for example, clear that a real-world head-up display comes with many limitations that require specific design adaptations that should be explicitly evaluated with respect to their impact [see, e.g., Trösterer et al. (2019)]. The results in this publication will advance questions on which information to show but are not meant to propose detailed ways how it should be shown.

An aspect that could not be investigated in this study for methodical reasons is the influence of the assistance systems on the acceptance of the driver and on the relationship between driver and passenger. It is possible, for example, that conflicts may be triggered because a driver does not keep the safety distance suggested by the “SD” system or does not react to the “Bu” signal from the passenger. It could be possible that ignoring the safety relevant information made for example by the “SD” or “At” system could even increase passenger discomfort in comparison to the same situations experienced without such a system. Agrawal and Peeta (2021), for example, showed in a study that unfavorable information could increase stress. This could also lead to more stress and discomfort for the passenger. Other negative effects of the assistance systems could be that the passenger distrusts the system states, or that information might be displayed too late. If the information is only available to the front passenger, as is the case with the “At” system, it could also be that the driver feels controlled by the passenger. Similarly, knowing about a system like “Bu,” the driver could ignore the system if (s) he does not trust or care about the passenger or the passenger could feel similar inhibitions as for direct communication. Combining the “Bu” systems with the functionality of “SD” System would make the feedback on the insufficient distance to the driver more anonymous and provide an additional trusted source to the signal for the driver. There could be arguments for and against showing assistance information only to the passengers or also to the driver, however, the effectiveness of the shared distance concept, revealing the driver’s comparator, might favor a joint visualization. It should also be mentioned that the driver could also have expectations such as additional support from the passenger when the passenger is provided with information by a passenger assistance system. These possible side effects on the driver and the relationship between the two vehicle occupants can be part of further research. Additionally, the study was conducted with a driver unfamiliar to the participant. Since interviews (Ittner et al., 2020) showed that passengers more frequently travel with known drivers it would be another interesting topic to investigate the effect of the assistance systems under these conditions.

When interpreting the results of the studies, the effect of the reduced sample size on the power of the statistical tests has to be considered. This aspect does reduce the probability of finding an effect that actually exists. It is possible that potential effects of the assistance systems on passenger discomfort were not detected due to the reduced sample size. However, due to the positive subjective evaluations of the participants, it is conceivable that a possible reducing effect of the systems on passenger discomfort was underestimated from the direction of the effect. This means, that especially with the additional supporting results of the subjective evaluations by the participants, basic statements can be made regarding passenger assistance systems and their ability to reduce passenger discomfort.

### 5.5. Conclusion

In conclusion, this work could show that it is possible to design a passenger assistance system that reduces discomfort. It also becomes clear that there is a lot of potential in taking the passenger more into account during the design process of assistance systems. Even rudimentary information, some of which is currently only displayed to the driver (e.g., drowsiness warning, distance indication with adaptive cruise control), could have positive effects on the passenger’s driving experience if it would also be available to them. Some of the presented results might not only be relevant for the further development of assistance systems in conventional vehicles but might also apply to settings with higher automation levels when the driver will also turn into a passenger of the vehicle. However, some further aspects need to be considered when developing passenger assistance systems. In general, the presented work highlights possibilities to increase the comfort of passengers beyond infotainment systems.

## Data availability statement

The raw data supporting the conclusions of this article will be made available by the authors, without undue reservation.

## Ethics statement

The studies involving human participants were reviewed and approved by own ethics committee following a strict ethics protocol when planning our studies with healthy participants. This ethics committee follows recommendations of the German Research Association (Deutsche Forschungsgemeinschaft, 2019). The patients/participants provided their written informed consent to participate in this study.

## Author contributions

SI, DM, TW, and MV: conceptualization and methodology. SI: software, formal analysis, investigation, writing – original draft preparation, visualization, and data curation. SI, DM, and TW: validation. SI and TW: resources. DM, TW, and MV: writing – review and editing. SI and TW: project administration. All authors have read and agreed to the published version of the manuscript.

## Funding

This study was conducted as part of a research program of the Honda Research Institute Europe GmbH.

## Conflict of interest

This study was conducted as part of a research program of the Honda Research Institute Europe GmbH. TW is employee of this company. He contributed to the design of the study, review and editing of the manuscript, and the decision to publish the results. SI and DM are employed by Wuerzburg Institute for Traffic Sciences GmbH.

The remaining authors declare that the research was conducted in the absence of any commercial or financial relationships that could be construed as a potential conflict of interest

## Publisher’s note

All claims expressed in this article are solely those of the authors and do not necessarily represent those of their affiliated organizations, or those of the publisher, the editors and the reviewers. Any product that may be evaluated in this article, or claim that may be made by its manufacturer, is not guaranteed or endorsed by the publisher.

## Footnotes

^1^https://wivw.de/en/silab
