# Supplementary material for: Development and evaluation of passenger assistance system concepts to reduce passenger discomfort
Source: Front Psychol. 2023 Feb 9;14:1024540. doi: 10.3389/fpsyg.2023.1024540 (PMC9947555; doi:10.3389/fpsyg.2023.1024540)
Supplement: Supplementary file 1 [file Table_1.docx]

# Supporting information

**S1 Table. Formulations of all investigated items.**

| Criticality | How safety critical was the situation? |
| --- | --- |
| Trust | How much did you trust the driver in that situation? |
| Discomfort | How uncomfortable did you feel as a passenger in the situation? |
| Estimate | How helpful was the assistance system to better assess the situation? |
| Exposed | How much did you feel exposed to the situation as a passenger? |
