# Supplementary material for: Development and evaluation of passenger assistance system concepts to reduce passenger discomfort
Source: Front Psychol. 2023 Feb 9;14:1024540. doi: 10.3389/fpsyg.2023.1024540 (PMC9947555; doi:10.3389/fpsyg.2023.1024540)
Supplement: Supplementary file 2 [file Table_2.docx]

# Supporting information

**S2 Table. Positive and negative statements per assistant system made in the post inquiry as a reason for the chosen helpfulness rating.**

|  | **Positive Statements** | | **Negative Statements** | |
| --- | --- | --- | --- | --- |
|  | **Reasons** | ***N* = 32** | **Reasons** | ***N* = 16** |
| **At** | The system gives you security/a good feeling when the driver is concentrated | 5 | Does not replace trust | 2 |
|  |  |  | Forces passenger to pay attention | 2 |
| **SD** | Better estimation of situation | 3 | No effect | 1 |
|  | The system gives you security | 3 |  |  |
|  | Certainty that nothing can happen | 1 |  |  |
|  | Support for the driver | 1 |  |  |
| **Bu** | Possibility to intervene | 3 | Negative effect/more anxiety | 2 |
|  | Discreet hint | 1 | Automatic system would be more effective | 1 |
|  | Driver behavior is confirmed | 1 | No help | 1 |
|  | Good for anxious passengers | 1 |  |  |
| **BI** | Certainty that everything in control | 2 | Irrelevant | 2 |
|  | Feedback of driver reaction | 2 | Negative effect/ more anxiety | 1 |
|  | Only useful if you do not know the driver | 1 |  |  |
|  | Supportive, if passenger recognizes situation possibly earlier | 1 |  |  |
| **PTHW** | Better estimation of (safety) distance | 4 | Senseless with preferred distance | 2 |
|  | More security/calming | 2 | Hard to switch off | 1 |
|  | Feeling less exposed | 1 | More useful for driver | 1 |
|  | | | | |
