# Supplementary material for: Development and evaluation of passenger assistance system concepts to reduce passenger discomfort
Source: Front Psychol. 2023 Feb 9;14:1024540. doi: 10.3389/fpsyg.2023.1024540 (PMC9947555; doi:10.3389/fpsyg.2023.1024540)
Supplement: Supplementary file 3 [file Table_3.docx]

# Supporting information

**S3 Table. Positive and negative reasons made in the post inquiry for the intention to use the experienced assistant system.**

|  | **Yes/Yes, but it depends on the driver or situation** | | **Maybe/I don’t know/No** | |
| --- | --- | --- | --- | --- |
|  | **Reasons** | ***N* = 33** | **Reasons** | ***N* = 10** |
| **At** | Depends on situation (dichter Verkehr, Nachtfahrt, Langstrecke) | 2 | No possibility of influence | 1 |
|  | When already built-in | 1 |  |  |
|  | The system provides security | 1 |  |  |
| **SD** | Provides more information to better assess the situation | 3 | Negative effects like need would arise to control the driver | 1 |
|  | When already built-in | 2 | The more thinking is taken away, the less you think for yourself. What if technology fails? | 1 |
|  | Depends on driver/situation | 2 |  |  |
|  | More trust in driver | 1 |  |  |
| **Bu** | Depends on the driver/situation | 2 | System actually not necessary, can also be said | 2 |
|  | More comfort | 1 |  |  |
|  | Discreet influence is comfortable | 1 |  |  |
|  | Support for the driver if you are attentive as a passenger | 1 |  |  |
|  | Would only use it if it is an automatic system | 1 |  |  |
| **BI** | Depends on driver | 1 | No added value | 1 |
|  | Permanent use | 1 | Would not use it | 1 |
|  | Matter of habit | 1 | I cannot judge because there was no dangerous situation | 1 |
|  | Safety and security | 1 |  |  |
|  | The system can give feedback when something needs to be said | 1 |  |  |
| **PTHW** | If already installed | 4 | No effect | 1 |
|  | If you don't trust the driver | 2 | Possibly yes, but do not activate actively | 1 |
|  | Visual feedback | 2 |  |  |
|  | Depends on situation | 2 |  |  |
|  | | | | |
