# Supplementary material for: Development and evaluation of passenger assistance system concepts to reduce passenger discomfort
Source: Front Psychol. 2023 Feb 9;14:1024540. doi: 10.3389/fpsyg.2023.1024540 (PMC9947555; doi:10.3389/fpsyg.2023.1024540)
Supplement: Supplementary file 4 [file Table_4.docx]

# Supporting information

**S4 Table. Distribution of participants across the categories of gender, age, passenger and driver experience in the subgroups.**

|  | **Gender** | |
| --- | --- | --- |
|  | **Female [N]** | **Male [N]** |
| **At** | 4 | 4 |
| **SD** | 4 | 5 |
| **Bu** | 4 | 4 |
| **BI** | 5 | 3 |
| **PTHW** | 5 | 3 |

|  | **Age** | | |
| --- | --- | --- | --- |
|  | **20-30 years [N]** | **30-60 years [N]** | **60 years < [N]** |
| **At** | 2 | 4 | 2 |
| **SD** | 2 | 4 | 2 |
| **Bu** | 3 | 3 | 2 |
| **BI** | 2 | 4 | 2 |
| **PTHW** | 2 | 4 | 2 |

|  | **Passenger Experience** | | | |
| --- | --- | --- | --- | --- |
|  | **3-5 x per week [N]** | **1-2 x per week [N]** | **1-3 x per month [N]** | **less than once per month [N]** |
| **At** | 2 | 3 | 1 | 2 |
| **SD** | 0 | 4 | 3 | 1 |
| **Bu** | 3 | 1 | 2 | 2 |
| **BI** | 1 | 3 | 4 | 0 |
| **PTHW** | 1 | 3 | 4 | 0 |

|  | **Driver Experience** | | | | |
| --- | --- | --- | --- | --- | --- |
|  | **(almost) daily [N]** | **3-5 x per week [N]** | **1-2 x per week [N]** | **1-3 x per month [N]** | **less than once per month [N]** |
| **At** | 6 | 2 | 0 | 0 | 0 |
| **SD** | 5 | 2 | 1 | 0 | 0 |
| **Bu** | 4 | 0 | 2 | 1 | 1 |
| **BI** | 7 | 1 | 0 | 0 | 0 |
| **PTHW** | 5 | 2 | 1 | 0 | 0 |
